# A Two-stage Dynamic Undesirable Data Envelopment Analysis Model Focused on Media Reports and the Impact on Energy and Health Efficiency

**DOI:** 10.3390/ijerph16091535

**Published:** 2019-04-30

**Authors:** Huaming Chen, Jia Liu, Ying Li, Yung-Ho Chiu, Tai-Yu Lin

**Affiliations:** 1 College of Literature and Journalism, Sichuan University, Wangjiang Road No.29, Chengdu 610064, China; chenhuaming@scu.edu.cn (H.C.); 2017321030051@stu.scu.edu.cn (J.L.); 2 Business School, Sichuan University, Wangjiang Road No. 29, Chengdu 610064, China; 3 Department of Economics, Soochow University, 56, Kueiyang St., Sec. 1, Taipei 100, Taiwan; echiu@scu.edu.tw (Y.-H.C.); eickyla@gmail.com (T.-Y.L.)

**Keywords:** media, public health, energy efficiency, environmental efficiency, Two-Stage Dynamic SBM model efficiency

## Abstract

Past research on energy and environmental issues in China has generally focused on energy and environmental efficiencies with no models having included the public health associations or the role of the media. Therefore, to fill this research gap, this paper used a modified Undesirable Dynamic Network model to analyze the efficiency of China’s energy, environment, health and media communications, from which it was found that the urban production efficiency stage was better than the health treatment stage, and that the energy efficiencies across the Chinese regions varied significantly, with only Beijing, Guangzhou, Lhasa and Nanning being found to have high efficiencies. Large urban gaps and low efficiencies were found for health expenditure, with the best performances being found in Fuzhou, Guangzhou, Haikou, Hefei, Nanning, and Urumqi. The regions with the best media communication efficiencies were Fuzhou, Guangzhou, Haikou, Hefei, Lhasa, Nanning and Urumqi, and the cities with the best respiratory disease efficiencies were Fuzhou, Guangzhou, Haikou, Lhasa, Nanning, Wuhan, Urumqi, Xian, and Yinchuan. Overall, significant efficiency improvements were needed in health expenditure and in particular in respiratory diseases as there were major differences across the country.

## 1. Introduction

In 2001, China’s GDP was 1,086.3 billion CNY, which after an average annual growth rate of 9.3% had risen to 827,121.7 billion CNY by 2017 [1]. This rapid rise in economic growth was mainly attributable to industrial sector development, for which coal, oil, natural gas and energy were the main driving forces. As 60% of China’s energy comes from coal fired power [2], significant quantities of sulphur dioxide, carbon monoxide, dust, nitrogen oxides, and carbon dioxide have been released into the air, which has led to a rise in the number of asthma and respiratory disease cases in Chinese cities, which has not only endangered citizen health but has also added to China’s public health expenditure. For instance, sulfur dioxide causes respiratory diseases such as bronchitis and lung disease, carbon monoxide causes feeling, understanding and memory losses, and nitrogen dioxide causes a decrease in lung functions. China is the world’s largest coal consumer, its third largest oil consumer, and its fourth largest natural gas consumer [2]. 

Therefore, China’s key current problems are focused on reducing air pollution and the commensurate health problems while maintaining economic development. As part of the 2015 Paris Agreement, China agreed to three major goals: (1) carbon dioxide emissions would reach a peak in 2030; (2) by 2030, CO_2_ emissions are to drop by 60% to 65% of 2005 levels; and (3) by 2030, non-fossil energy will account for 20% of China’s energy consumption [2]. At the same time, the Chinese government issued the “Air Pollution Prevention and Control Action Plan”, with the goal of reducing PM_2.5_ emissions by 50% of 2015 levels by 2040. In addition to government policies, in recent years, due to technological developments, the media has increased its influence on Chinese residents, which has led some to believe that the media could be used to encourage public air pollution monitoring and to reduce the incidence of air pollution related diseases [3].

Generally, energy and environmental pollution research has focused on energy and environmental efficiency analyses [4,5,6,7,8,9,10,11,12,13,14,15,16,17,18,19,20,21], with most studies having examined the energy and CO_2_ efficiencies in China’s eastern and western provinces or cities, from which it has been found that there were significant improvements needed in China’s CO_2_ efficiency and that the energy efficiency in eastern China was better than in the midwest. Research has also explored the effects of air pollutants on human health [22,23,24,25,26,27,28,29,30,31,32,33,34,35,36,37,38], methods for reducing the health effects of air pollution [39,40], and the long-term health effects of PM2.5 exposure, such as cardiovascular and respiratory diseases and death.The relationship between the mass media and public health [41,42,43,44,45,46,47,48], and the effect of media reports on air pollution reductions and associated public health problems [49,50,51,52,53] has been found to enhance public understanding and attention to air pollution and reduce the negative health impacts of air pollution.

As most of the above research has only focused on a single issue such as environmental efficiency, energy efficiency, the effects of pollution on human health, or the effects of media reports on pollution reduction, there has been no integration of the environmental, human health and media report efficiency measures. However, in more recent studies, Network Data Envelopment Analysis (DEA) has been adopted to analyze both the production and pollution treatment stages. For example, Fare et al. [54], Hampf [55], Lozanzo [56] and Wu et al. [8] used a two-stage DEA or Network slacks-Based Measure (NSBM) to examine a production stage and a pollution treatment stage. To analyze the production, waste water, and waste gas efficiencies in 30 Chinese regions, Wu et al. [57] used a two-stage network in which the first phase was the production system and the second phase was the disposal system. The two-stage DEA analysis used environmental protection inputs and energy consumption management to reduce carbon dioxide and air pollutant emissions and maintain production efficiency.

However, there has been less research jointly focused on the associations between energy, environmental pollution, health and the media. In the production stage, while the labor and energy inputs generate good GDP output, it also outputs air pollutants. Therefore, the undesirable intermediate output (AQI and CO_2_) from the production stage (first stage) could be regarded as the input resource in terms of illness or death for the health treatment stage (second stage). In the second stage, the government health expenditures and media reports could be used to reduce disease prevalence. Therefore, the relationship between energy, health and media coverage can be constructed using a two-stage DEA model. The theoretical framework for this study is based on previous study results and the following assumptions:

(1) While fossil energy consumption and labor input contribute to economic growth, they also result in environmental issues such as air pollution, carbon emissions and ecological damage.
(2) Air pollutants have a heavy impact on individual respiratory and heart functions and lead to higher social and individual health costs.
(3) Public media reports positively impact public awareness of air pollution and its impact on the environment and human health.
(4) All the above factors and the relative impact on the environment and public health could lead to greater long term health and environmental rectification expenditure.

**Figure 1:**
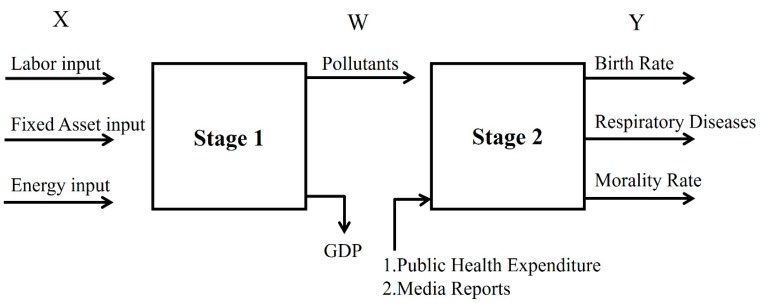
Process.

Based on these assumptions, this study employed network data development analysis to explore the air pollutant effects on respiratory diseases, birth and mortality rates, and public health treatment and public media efficiencies. Based on the results, policy and managerial suggestions are also given (see Figure 1).

This research analyzed the impact of government spending on health care and media coverage through an energy and environmental analysis. A two stage DEA model was applied to analyze the energy, health and media reporting efficiencies in 31 mainland Chinese cities. This model construction not only maintained the existing production efficiency, but also considered the protection of citizen health.

As most environmental pollution and energy efficiency analyses have usually been conducted using a static DEA), the results have given little guidance on energy and environmental sustainability. Further, traditional one-stage DEA energy and environmental efficiency analyses have also failed to consider public health or media issues. Therefore, to extend the current restricted research framework, this study used a Modified Undesirable Dynamic Network model to explore China’s energy, environment, health, and media communication efficiencies. 

This article has two main contributions. First, in addition to exploring traditional energy and environmental efficiency, health and media communication factors were included in the model to comprehensively explore the energy, environment, health and media communication associations. Second, using the modified Undesirable Dynamic Network model, the disadvantages associated with static analyses were avoided. 

Data from 2013–2016 for 31 Chinese cities were extracted and analyzed. Production was taken as the first stage, which had labor and energy consumption as the inputs and GDP as the output. The link between the production stage and the health treatment stage variables were CO_2_ emissions and the AQI, and health treatment was the second stage, which had health expenditure and media reports as the inputs, birth rate, respiratory disease and death rate as the outputs, and fixed assets as the carry over. The remainder of this paper is organized as follows: Section 2 gives the literature review, Section 3 describes the research method, Section 4 gives the empirical results and discussion, and Section 5 gives the conclusions.

## 2. Literature Review

There have been three main research directions in analyses of energy, environment, health, and the media. The first area has focused on energy and environmental efficiency analyses. For example, Hu and Wang [4] analyzed China’s energy efficiency using a modified DEA model and found that China’s rapid economic development had led to energy efficiency improvements, and Song et al. [5] used a Super-SBM model to measure and calculate the energy efficiency in BRICS countries, finding that they were less energy efficient and that as the relationships between energy efficiency and carbon emissions varied from country to country due to the different energy structures, energy policies needed to be developed based on each country’s unique conditions. Wang et al. [6] used a multi-directional efficiency analysis (MEA) rather than the traditional linear DEA to study regional energy and emission efficiencies in China, and found that the eastern region was more efficient than the central and western regions. Wang et al. [7] researched the carbon dioxide emissions performance of various provinces in China and found that the CO_2_ emissions in the southeastern coastal areas were relatively high, and the CO_2_ emissions in the inland central and western regions were relatively low. Wu et al. [8] assessed China’s energy conservation and emissions reduction efficiency, and found that: (1) eastern China had the best energy conservation and emissions reduction; (2) the central region had superior production efficiency to the western region but the western region had better processing efficiency; (3), and China’s overall energy conservation and emissions reduction efficiencies were stable and pollution control efficiency was rising. Li and Du [9] analyzed the impact of marketization on China’s energy and carbon emissions efficiency and found that overall, the energy use and CO_2_ emissions performances were poor, but that they were better in the eastern region than in the central west. Meng et al. [10] evaluated China’s energy and carbon discharge efficiency (EE&CE), finding that eastern China had the highest EE&CE and central China had the lowest. Yao et al. [11] used panel data and a meta-frontier non-radial Malmquist CO_2_ emissions performance index (MNMCPI) to examine China’s provincial industrial sector from 1998 to 2011 and estimate China’s carbon dioxide emissions efficiency, finding that the annual industrial sector carbon dioxide emissions growth rate was 5.53%, and that the average industrial sector carbon dioxide emissions in the eastern, central and western regions had declined. Jebali et al. [12] researched the energy efficiency of Mediterranean countries from 2009-2012 and found that it was very high. Abbas et al. [13] reviewed different DEA models in terms of energy efficiency development. Qin et al. [14] assessed the energy efficiency of China’s coastal areas from 2000 to 2012, and found that they had the following characteristics: (1) economic development was positively correlated with energy efficiency performance; (2) except for Beijing and Hainan, energy efficiency had generally declined; (3) Energy efficiency had improved in the Bohai Economic Zone; (4) the Malmquist-Luenberger productivity growth rate was overestimated; and (5) the main obstacles to energy efficiency were related to the lack of technological improvements, scale efficiencies and adequate management standards. Feng et al. [15] analyzed China’s carbon dioxide emissions efficiency, and found that there was low technical, management, and carbon dioxide emissions efficiencies. Hu et al. [16] used a congestion total-factor energy efficiency model to analyze electricity and lighting electricity consumption and gasoline and diesel sales in Taiwan’s 20 administrative regions from 2004 to 2013. Zeng et al. [17] used structural VAR models and Beijing carbon emission quotas to study the dynamic relationships between carbon credits, economic development and energy prices, finding that a standard deviation in coal price increases would lead to an initial increase of about 0.1% in Beijing’s carbon price, and that Beijing’s carbon allowance price was mainly affected by the historical price. Mehmeti et al. [18] analyzed fuel battery energy efficiency. As the pulp and paper industry is one of the largest energy consumers, greenhouse gas (GHG), and pollutant emitters, Sun et al. [19] examined the life cycle environmental impacts of pulp and paper systems in 45 papermaking and 18 pulping samples, and found that 1 tonne of paper emitted on average about 950 kg of carbon dioxide (CO_2_ equivalent) of greenhouse gas emissions, and that there were significant differences between countries and the pulp and paper categories, with the main factor affecting greenhouse gas emissions being energy use. Li et al. [20] summarized the policies affecting the development of the nonferrous metals industry (NMI) in China, and concluded that if the central government adhered to the “13th Five-Year Plan” (2016–2020) carbon dioxide emission reduction policy, the copper, lead and zinc industries would reach their upper emissions limits by 2030. Zeng et al. [21] assessed the investment efficiency of China’s new energy industry, and found that between 2012 and 2015, the overall investment efficiency of the new energy industry was relatively low, with an average total technical efficiency of 44%, a pure technical efficiency of 48%, and a scale efficiency of 90%. Some other studies employed Network DEA to integrate the production stages and pollution treatment stages to analyze energy efficiency and government pollution treatment. [8,54,55,56,57].

The second research area has been focused on analyses of the impact of air pollution on human health. For example, Oakes et al. [22] studied air pollution and human health risk assessments, and Schiavon et al. [23] simulated the effects of NOx emissions from urban road traffic on human health using the COPERT algorithm and the AUSTAL2000 dispersion model and found that high-concentration emissions from the streets had an impact on the human body. Schiavon et al. [24] explored the ability of air quality monitoring stations to detect potential crises and found that they may be inefficient, Fischer [25] explored the relationship between PM_10_ and NO_2_ mortality in the population over 30 years old in the Netherlands, and Kelly and Fussell [26] found that particulate matter (PM) had an impact on health. Lelieveld et al. [27] examined the effects of outdoor air pollution (mainly PM_2.5_), finding that it caused 3.3% of global mortality per year, Lu et al. [28] researched the relationship between PM2.5 and disease, finding that an increase of 10 μg/m^3^ in PM_2.5_ resulted in an overall non-accidental associated mortality rate increase of 0.40%, a cardiovascular disease-induced mortality increase of 0.63%, and a respiratory disease mortality rate increase of 0.75%, and Pope et al. [29] demonstrated that fine particulate matter increased the risk of disease and mortality. Tainio et al. [30] found that when there was a PM_2.5_ of 100 μg/m^3^, riding for one and a half hours or walking for more than 10 hours a day could cause more damage than benefits, Khafaie et al. [31] also found that exposure to outdoor air pollution had an adverse effect on health, and Pannullo et al. [32] investigated the relationship between CO_2_ concentrations in midwest Scotland and cardiopulmonary respiratory mortality from 2006–2012. Johansson et al. [33] found that a NOx concentration drop of 10 μg/m^3^ in the most densely populated areas of central Stockholm reduced the relative risk of mortality by 8%, Cohen et al. [34] found that the mortality from PM_2.5_ increased from 35 million in 1990 to 42 million in 2015, and Newell et al. [35] found that air pollution exposure was associated with cardiopulmonary disease and mortality in low- and middle-income countries. Zigler et al. [36] used causal inference methods and a spatially hierarchical regression model to investigate the environmental impact of environmental particulate matter in 2005, Kinney [37] found that ozone and PM2.5 emissions increased at higher ambient temperatures, Lua et al. [38] demonstrated that the health cost-effectiveness of PM2.5 pollution reduction in 2017 was $193,800 in China, accounting for 1.58% of the country’s total GDP, Li et al. [39] concluded that an emissions trading system could reduce the economic losses caused by air pollution on public health, and Rich [40] reviewed the statistical methods used to assess the relationships between public health and air pollution.

The third main research area has been analyses of mass media and public health. For example, Griffiths and Knutson [41] concluded that mass communication could be used successfully to promote public health, Wilde [42] reviewed media communication, health, and safety relevance, and Bertrand et al. [43] studied the effect of 24 mass media types on changing the public’s knowledge and attitudes toward the human immunodeficiency virus (HIV). Maloney and Cappella [44] explored how cigarette advertising could encourage smokers or quitters to smoke, Noar [45] explored how mass media activities could increase health knowledge, and Costa and Kahn [46] studied newspaper and public health issues from the late 19th century to the early 20th century. Gonsalves et al. [47] concluded that mass communication increased female awareness of cardiovascular disease prevention and reduced female cardiovascular disease incidence, Mirte et al. [48] assessed the relationship between UK mass media spending and smoking cessation attempts, smoking cessation, and smoking prevalence rates from 2008 to 2016 and found that the higher the monthly expenditure on tobacco control mass media campaigns in the UK, the higher the smoking cessation success rate, and Mayar [49] analyzed the relationship between air pollution and asthma in The New York Times, The Los Angeles Times and The Washington Post. Jiang et al. [50] demonstrated that social media information was highly correlated with the air quality index, Wang [51] found that social media data could increase existing air pollution monitoring data, and Elliot et al. [3] demonstrated that mass media coverage increased health care online searches. Murukutla et al. [52] commented on India’s news reports and policy advances on air pollution, and Schwabe [53] found that the media could increase the public’s influence on air pollution policy.

Table 1 outlines the three main previous research areas: energy and its impact on environment, energy and its impact on public health, and media reports and the impact on public health or pollution policies. Even though traditional DEA methods have focused on one or two of these research areas, few studies have examined these areas in concert using scientific methods, or examined the impact of both air pollutant emissions and health treatment together with media reports. Therefore, to fill this research gap, this paper used a modified Undesirable Dynamic Network model to explore the energy, the environment, health, and media communication efficiencies in 31 Chinese cities. 

## 3. Research Method

DEA is generally used to assess multiple orientations and multiple decision-making priorities through the establishment of an efficiency index that includes input and output variable data for each associated decision making unit (DMU), and uses linear programming to develop an efficiency frontier. The relative efficiency of each individual DMU is then determined by its distance from the efficiency frontier.

Farrell [58] first proposed the related concepts, after which Charnes et al. [59] extended the DEA concept, and then Banker et al. [60] changed the assumption and designed the BCC model (named after Banker, Charnes, and Cooper). Andersen and Petersen [61] then proposed a modified DEA (super efficiency model) based on a constant return to scale to resolve the problems when a super efficiency value ≧1 could appear. However, as Thrall [62] found that the super-efficiency model had problems that could not be estimated in the case of variable returns to scale, Tone [63] proposed an efficiency estimation model (SBM model) based on the difference variable and then a Slacks-Based Measure (SBM) that was non-radial and considered the differences between the inputs and outputs. Following these innovations, Färe et al. [64] then proposed Network Data Envelopment Analysis (Network DEA) to resolve the problem of the "black box" in the production process.

Tone and Tsutsui [65] then developed a weighted slack-based measures network data envelopment analysis model in which the links between the various departments of each DMU were used as the basis for the Network DEA model analysis, and then the SBM model used to find the best solution, which overcame the problems with traditional DEA. As company operations span many periods, a Dynamic DEA model was then developed so that departmental efficiencies could be assessed over time; that is, a combination of the Network DEA and the Dynamic DEA was necessary. Consequently, Tone and Tsutsui [66] proposed the weighted slack-based measures (Dynamic Network DEA model), in which the links between the various DMU departments (or sub DMUs) were used as the basis for the analysis of the Network DEA model, and in which each Sub-DMU and the linked carry-over activities were assessed as (1) desirable, (2) undesirable, (3) discretionary, or (4) nondiscretionary. The dynamic DEA model analysis was therefore divided into input-oriented, output-oriented, and non-oriented, and the SBM model then used to find the best solution.

Even though Tone and Tsutsui’s [66] Dynamic Network DEA model set the undesirable output as carry-over activities, the output did not consider the undesirable output variable. This paper, therefore, corrects Tone and Tsutsui’s [66] model, by considering the undesirable output in the Dynamic Network DEA model; in this paper, called the modified Undesirable Dynamic Network model.

### 3.1. Modified Undesirable Dynamic Network Model

Most existing research has focused on the effects of energy, the environment, health, and media reports on environmental pollution and personal awareness, and the impact of media reports on public health awareness. However, there have been few systematic scientific studies on the effects of energy, the environment, health, and media reports on public health.

**Figure 2:**
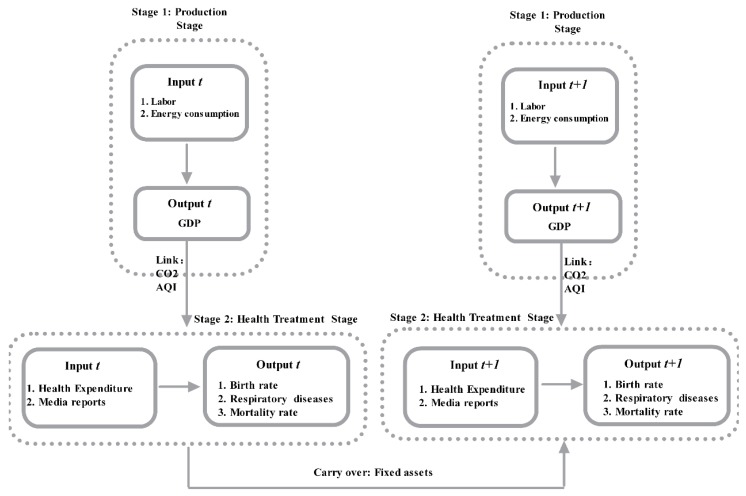
Undesirable Dynamic Network Model.

This study collected data from 31 Chinese cities, and adjusted Tone and Tsutsui’s [66] Dynamic Network model to include undesirable output; therefore, the model used for the analyses was a modified Undesirable Dynamic Network model, in which there were two stages; the first stage inputs were labor and energy consumption and the output was GDP, with the link variables to the second stage being CO_2_ emissions and the AQI, and the second stage inputs were health expenditure and media reports, and the outputs were birth rate, respiratory diseases, and mortality rate, with the carry over being fixed assets investment. Figure 2 shows the framework for the modified Undesirable Dynamic Network model inter-temporal efficiency measurements and variables. 

The Modified Undesirable Dynamic Network Model. was designed as follows:

Suppose there are *n DMU*s (*j* = 1,…,*n*), with each having *k* divisions (*k* = 1,…,*K*), and *T* time periods (*t* = 1,…,*T*). Each of the *DMU*s has an input and output at time period *t* and a carryover (link) to the next *t*+1 time period.

Set *m_k_* and *r_k_* to represent the inputs and outputs in each division *K*, with (*k,h*)*i* representing divisions *k* to *h* and *L_hk_* being the *k* and *h* division set, The inputs, outputs, links, and carryover definitions are outlined in the following paragraphs. The following is the non-oriented model: 

(a) Objective function

Overall efficiency:

(1) $$\theta_{0}^{*}=\min\frac{\sum_{t=1}^{T}W^{t}\left[\sum_{k=1}^{K}W^{k}\left[1-\frac{1}{m_{k}+linkin_{k}}(\sum_{i=1}^{m_{k}}\frac{S_{iok}^{t-}}{x_{iok}^{t}}+\sum_{{\left(kh\right)}_{l}=1}^{linkin_{k}}\frac{s_{o{\left(kh\right)}_{l}in}^{t}}{z_{o{\left(kh\right)}_{l}in}^{t}})\right]\right]}{\sum_{t=1}^{T}W^{t}\left[\sum_{k=1}^{K}W^{k}\left[1+\frac{1}{r_{1k}+r_{2k}+ngood_{k}}(\sum_{r=1}^{r_{1k}}\frac{s_{rokgood}^{t+}}{y_{rokgood}^{t}}+\;\sum_{r=1}^{r_{2k}}\frac{s_{rokbad}^{t-}}{y_{rokbad}^{t}}+\sum_{k_{l}}^{ngood_{k}}\frac{s_{ok_{l}good}^{\left(t,t+1\right)}}{z_{ok_{l}good}^{\left(t,t+1\right)}})\right]\right]}$$

Subject to:

$x_{ok}^{t}=X_{k}^{t}\lambda_{k}^{t}+s_{ko}^{t-}(\forall k,\forall t)$
$y_{okgood}^{t}=Y_{kgood}^{t}\lambda_{k}^{t}-s_{kogood}^{t+}(\forall k,\forall t)$
$y_{okbad}^{t}=Y_{kbad}^{t}\lambda_{k}^{t}+s_{kobad}^{t-}(\forall k,\forall t)$
$e\lambda_{k}^{t}=1(\forall k,\forall t)$
$\lambda_{k}^{t}\geq 0,s_{ko}^{t-}\geq 0,s_{kogood}^{t+}\geq 0,s_{kobad}^{t-}\geq 0,(\forall k,\forall t)$
$Z_{o(kh)in}^{t}=Z_{(kh)in}^{t}\lambda_{k}^{t}+S_{o(kh)in}^{t}((kh)in=1,\ldots ,linkin_{k})$
$\sum_{j=1}^{n}z_{jk_{1}\alpha }^{\left(t,(t+1)\right)}\lambda_{jk}^{t}=\sum_{j=1}^{n}z_{jk_{1}\alpha }^{\left(t,(t+1)\right)}\lambda_{jk}^{t+1}(\forall k;\forall k_{l};t=1,\ldots ,T-1)$
$Z_{ok_{l}good}^{\left(t,(t+1)\right)}={\sum_{j=1}^{n}z}_{jk_{l}good}^{\left(t,(t+1)\right)}\lambda_{jk}^{t}-s_{ok_{l}good}^{\left(t,(t+1)\right)}k_{l}=1,\ldots ,ngood_{k};\forall k;\forall t)$
$s_{ok_{l}good}^{\left(t,(t+1)\right)}\geq 0,\left(\forall k_{l};\forall t\right)$

(b) Period and division efficiencies

Period and division efficiencies are as follows:

(b1) Period efficiency:
  (2) $$\partial_{0}^{*}=\min\frac{\sum_{k=1}^{K}W^{k}\left[1-\frac{1}{m_{k}+linkin_{k}}(\sum_{i=1}^{m_{k}}\frac{S_{iok}^{t-}}{x_{iok}^{t}}+\sum_{{\left(kh\right)}_{l}=1}^{linkin_{k}}\frac{s_{o{\left(kh\right)}_{l}in}^{t}}{z_{o{\left(kh\right)}_{l}in}^{t}})\right]}{\sum_{k=1}^{K}W^{k}\left[1+\frac{1}{r_{1k}+r_{2k}+ngood_{k}}(\sum_{r=1}^{r_{1k}}\frac{s_{rokgood}^{t+}}{y_{rokgood}^{t}}+\;\sum_{r=1}^{r_{2k}}\frac{s_{rokbad}^{t-}}{y_{rokbad}^{t}}+\sum_{k_{l}}^{ngood_{k}}\frac{s_{ok_{l}good}^{\left(t,t+1\right)}}{z_{ok_{l}good}^{\left(t,t+1\right)}})\right]}$$
(b2) Division efficiency:
  (3) $$\varphi_{0}^{*}=\min\frac{\sum_{t=1}^{T}W^{t}\left[1-\frac{1}{m_{k}+linkin_{k}}(\sum_{i=1}^{m_{k}}\frac{S_{iok}^{t-}}{x_{iok}^{t}}+\sum_{{\left(kh\right)}_{l}=1}^{linkin_{k}}\frac{s_{o{\left(kh\right)}_{l}in}^{t}}{z_{o{\left(kh\right)}_{l}in}^{t}})\right]}{\sum_{t=1}^{T}W^{t}\;\left[1+\frac{1}{r_{1k}+r_{2k}+ngood_{k}}(\sum_{r=1}^{r_{1k}}\frac{s_{rokgood}^{t+}}{y_{rokgood}^{t}}+\;\sum_{r=1}^{r_{2k}}\frac{s_{rokbad}^{t-}}{y_{rokbad}^{t}}+\sum_{k_{l}}^{ngood_{k}}\frac{s_{ok_{l}good}^{\left(t,t+1\right)}}{z_{ok_{l}good}^{\left(t,t+1\right)}})\right]}$$
(b3) Division period efficiency:
  (4) $$\rho_{0}^{*}=\min\frac{1-\frac{1}{m_{k}+linkin_{k}}(\sum_{i=1}^{m_{k}}\frac{S_{iok}^{t-}}{x_{iok}^{t}}+\sum_{{\left(kh\right)}_{l}=1}^{linkin_{k}}\frac{s_{o{\left(kh\right)}_{l}in}^{t}}{z_{o{\left(kh\right)}_{l}in}^{t}})}{1+\frac{1}{r_{1k}+r_{2k}+ngood_{k}}(\sum_{r=1}^{r_{1k}}\frac{s_{rokgood}^{t+}}{y_{rokgood}^{t}}+\;\sum_{r=1}^{r_{2k}}\frac{s_{rokbad}^{t-}}{y_{rokbad}^{t}}+\sum_{k_{l}}^{ngood_{k}}\frac{s_{ok_{l}good}^{\left(t,t+1\right)}}{z_{ok_{l}good}^{\left(t,t+1\right)}})}$$

From the above, the overall efficiency, period efficiency, division efficiency and division period efficiency were obtained for 31 Chinese cities from 2013–2016.

### 3.2. Fixed Assets, Labor, Energy Consumption, Gdp, Health Expenditure, Birth Rate, Respiratory Diseases and Mortality Rate Efficiencies

Hu and Wang’s [4] total-factor energy efficiency index was followed to overcome any possible bias in the traditional energy efficiency indicators. There were eight key features in this efficiency study: labor, energy consumption, GDP, health expenditure, media, birth rate, respiratory diseases, and mortality rate efficiency.

In this study, “I” represented area and “t” represented time. In the following input and output definitions, if the target input was equal to the actual input, then the efficiency equaled 1 and was deemed efficient; however, if the target input did not equal the actual input, then the efficiency was less than 1 and was deemed inefficient.

*The first stage: production stage*

Input variables:
Labor efficiency = $\frac{\mathrm{target}\;\mathrm{labor}\;\mathrm{input}\;\left(i,\;t\right)}{\mathrm{actual}\;\mathrm{labor}\;\mathrm{input}\;\left(i,\;t\right)}$
Energy consumption efficiency = $\frac{\mathrm{target}\;\mathrm{energy}\;\mathrm{input}\;\left(i,\;t\right)}{\mathrm{actual}\;\mathrm{energy}\;\mathrm{input}\;\left(i,\;t\right)}$
Output variables:
Desirable output (GDP):
GDP efficiency = $\frac{\mathrm{Actual}\;\mathrm{desirableGDP}\;\mathrm{output}\;\left(i,\;t\right)}{\mathrm{Target}\;\mathrm{desirable}\;\mathrm{GDP}\;\mathrm{output}\;\left(i,\;t\right)}\;$

*The second stage: the health treatment stage*

Input variables
Media efficiency = $\frac{\mathrm{target}\;\mathrm{media}\;\mathrm{input}\;\left(i,\;t\right)}{\mathrm{actual}\;\mathrm{media}\;\mathrm{input}\;\left(i,\;t\right)}$
Health Expenditure efficiency = $\frac{\mathrm{target}\;\mathrm{health}\;\mathrm{expenditure}\;\mathrm{input}\;\left(i,\;t\right)}{\mathrm{actual}\;\mathrm{health}\;\mathrm{expenditure}\;\mathrm{input}\;\left(i,\;t\right)}$
Output variables:
Birth Rate efficiency = $\frac{\mathrm{actual}\;\mathrm{birth}\;\mathrm{rate}\;\mathrm{output}\;\left(i,\;t\right)}{\mathrm{target}\;\mathrm{birth}\;\mathrm{rate}\;\mathrm{output}\;\left(i,\;t\right)}$
Respiratory Diseases efficiency = $\frac{\mathrm{target}\;\mathrm{undesirable}\;\mathrm{respiratory}\;\mathrm{diseases}\;\mathrm{output}\;\left(i,\;t\right)}{\mathrm{actual}\;\mathrm{undesirable}\;\mathrm{respiratory}\;\mathrm{diseases}\;\mathrm{output}\;\left(i,\;t\right)}$
Mortality Rate efficiency =$\frac{\mathrm{Target}\;\mathrm{undesirable}\;\mathrm{mortality}\;\mathrm{rate}\;\mathrm{output}\;\left(i,\;t\right)}{\mathrm{Actual}\;\mathrm{undesirable}\;\mathrm{mortality}\;\mathrm{rate}\;\mathrm{output}\;\left(i,\;t\right)}$

In the first stage, if the target labor and energy environmental inputs equal the actual inputs, then the efficiencies are equal to 1, indicating overall efficiency; however, if the target labor and energy environmental inputs are less than the actual inputs, then the efficiencies are less than 1, indicating overall inefficiency. 

If the target desirable GDP output is equal to the actual desirable GDP output, then the GDP efficiency equals 1, indicating overall efficiency. If the actual desirable GDP output is less than the target desirable GDP output, then the GDP efficiency is less than 1, indicating overall inefficiency. 

In the second stage, if the target health expenditure and media inputs equal the actual inputs, then the efficiencies are equal to 1, indicating overall efficiency; however, if the target health expenditure and media inputs are less than the actual inputs, then the efficiencies are less than 1, indicating overall inefficiency.

If the target desirable birth rate output is equal to the actual desirable birth rate output, then the birth rate efficiency equals 1, indicating overall efficiency. If the actual desirable birth rate output is less than the target desirable birth rate output, then the birth rate efficiency is less than 1, indicating overall inefficiency. 

If the target undesirable respiratory disease and mortality rate outputs are equal to the actual undesirable outputs, then the efficiencies equal 1, indicating overall efficiency; however, if the target undesirable outputs are less than the actual undesirable outputs, then the efficiencies are less than 1, indicating overall inefficiency.

## 4. Empirical study

### 4.1. Data sources and Description

This study used panel data from 2013 to 2016 from 31 of the most developed cities in eastern and western China. The data were extracted from the Statistical Yearbook of China, the Demographics and Employment Statistical Yearbook of China [1], and the City Statistical Yearbooks. Air pollutant data were collected from China Environmental and Protection Bureau Annual Reports [67] and the China Environmental Statistical Yearbooks [68]. 

As the 31 sample cities varied in population, industry aggregation, natural resources, meteorology and geology, they were seen to be representative of the general situation in China.

Table 2 outlines the variables in each stage.

*The first stage: production stage*

Input variables:
Labor input: employees in each city at the end of each year. Unit: person.
Energy consumption: total energy consumption in each city. Unit: 100 million Tonnes.
Output variables:
Desirable output (GDP): GDP in each city each year. Unit: 100 million CNY.
Link Production Stage and health stage variables:
CO_2_: CO_2_ emissions in each city each year. Unit Tonnes
AQI: average annual air quality Index (AQI), which is the measured concentration of particulate matter (PM_2.5_, PM_10_), sulfur dioxide (SO_2_), and Nitrogen.

*The second stage: the health treatment stage*

Input variables:
Health expenditure: total annual health Expenditure. Unit:100 million CNY
Media reports: annual number of news stories in the People’s Daily Online and Xinhuanet Media’s official website on “province + air pollution”. The reason for choosing these two official news sites was that they both publish a great deal of news that reflects the national and provincial government perspectives.
Output variables:
Birth rate, Respiratory Diseases, Mortality Rate
Carry over:
Fixed assets: fixed assets investment in each city. Unit: 100 million CNY

### 4.2. Input-Output Index Statistical Analysis

**Figure 3:**
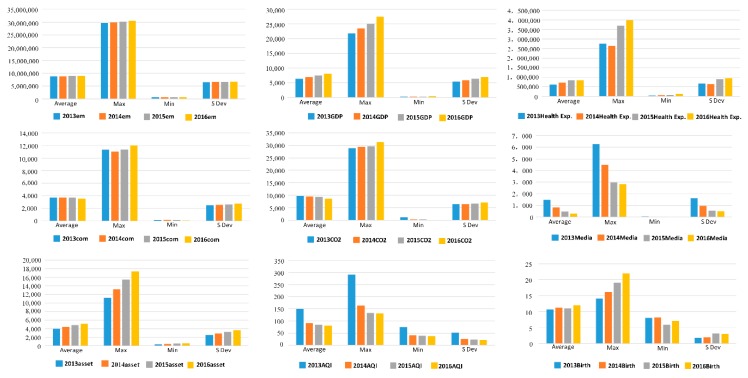
Input-output statistics.

Figure 3 gives a statistical picture of the overall inputs and outputs over the study period. As can be seen, the maximum and minimum labor values were slowly rising due to the slow population growth in China over the past few years, the maximum energy consumption declined from 2014 to 2015, but rose to a new high in 2016 and 2017, and the average reached its highest level in 2014, then began to decline in 2015 and fell below the 2015 level in 2016.

The maximum fixed assets growth was significant, but the increases in the minimum and average values were slower. The maximum GDP value increased significantly, the average value showed a steady increase, and the minimum value fluctuated, with the value falling slightly in 2015 and slightly increasing in 2016.

The health expenditure maximum fluctuated, falling in 2014, increasing in 2015, and reaching a new high in 2016, and the average and minimum values also fluctuated, but the volatility was relatively flat.

The media report maximum declined significantly in 2013, 2014 and 2015 and declined marginally in 2016, and the average value and the minimum value also declined.

The birth rate maximum was rising throughout the period, the minimum value rose in the first 2 years, decreased in 2015, and rose slightly in 2016, and the average value rose in 2014, declined in 2015, and reached its highest point in 2016.

### 4.3. Total Annual Efficiency Scores 

Table 3 shows the overall efficiencies in each city from 2013 to 2016. An overall efficiency of 1 in all four years was achieved by Guangzhou and Lhasa, and Nanning’s efficiency was 1 in the first three years and slightly above 0.8 in the last year. There were, however, overall efficiency improvements needed in the other cities.

While the efficiencies in Fuzhou, Haikou, Hefei, Wuhan, Urumqi, and Xian were above 0.6 but below 0.9, the overall efficiencies in the other 23 cities were all below 0.6. Beijing, Changchun, Chengdu, Guiyang, Harbin, Haikou, Huhehot, Nanchang, Nanjing, Shijiazhuang, Taiyuan, Wuhan, Urumqi, Xian, Xining, Yinchuan, and Zhengzhou all had falling efficiencies over the study period. In particular, Beijing’s efficiency declined from 1 in 2013 to 0.95 in 2014, to 0.6 in 2015, to 0.5 in 2016, Guiyang’s overall efficiency fell from 0.5 in 2013 to 0.3 in 2016, and Wuhan’s fell from 0.9 in 2013 to nearly 0.5 in 2016. The overall efficiencies in the other 12 cities fluctuated down.

The overall efficiencies in Changsha, Hangzhou, Jinan, Lanzhou, and Shanghai, however, increased over the study period. In particular, Jinan’s had the largest increase, from close to 0.4 in 2013 to 1 in 2016, and Shanghai’s rose from 0.5 in 2013 to 1 in 2015 and 2016. Therefore, the overall efficiency in these cities continued to improve.

### 4.4. Annual Efficiency Analysis in Each Stage

**Figure 4:**
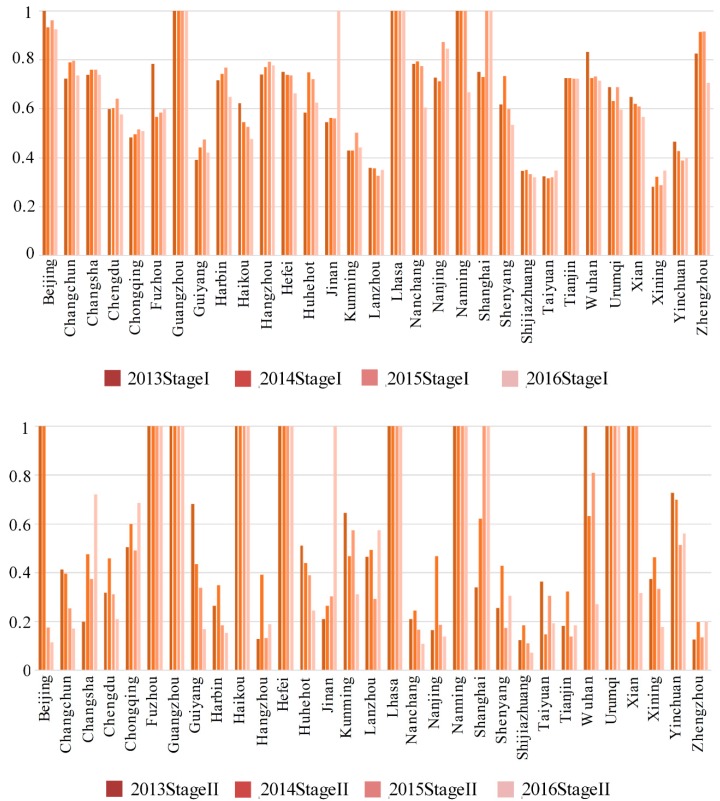
Two stage efficiency by city from 2013–2016.

Table 4 and Figure 4 show the two-stage efficiencies for all cities from 2013 to 2016. Most cities had better efficiency in the first stage than in the second stage, with the differences between the cities being significant in the second stage.

In the first stage, both Guangzhou and Lhasa had efficiencies of 1 in all four years, and Nanning had an efficiency of 1 for the first three years, but fell to only 0.6 in the final year. Shanghai’s first stage efficiency was lower than 0.7 in the first two years, and rose to 1 in 2015 and 2016, and Beijing’s was 1 in 2013 but dropped to around 0.9 over the next four years. However, 24 cities had first stage efficiencies below 0.8. In particular, Lanzhou, Shijiazhuang, Taiyuan, and Xining had first stage efficiencies lower than 0.4 in all four years, with the highest being slightly above 0.3.

However, Changchun, Chongqing, Guiyang, Hangzhou, Huhehot, Jinan, Kunming, Nanjing, Shanghai, Taiyuan, and Xining had increasing first stage efficiencies. While most cities only experienced small first stage efficiency increases, Jinan, Shanghai, and Nanjing had large increases, with Jinan’s rising from less than 0.6 in the first three years, Shanghai’s rising from 0.7 in the first two years and rising to 1 in 2015 and 2016, and Nanjing’s rising from 0.7 in the first two years to above 0.8 in the following years.

The differences between the cities in the second stage were greater than in the first stage. Fuzhou, Guangzhou, Haikou, Hebei, Lhasa, Nanning, and Urumqi’s second stage efficiencies were 1 in all four years, Beijing scored 1 in the first 2 years, but the efficiency fell to only 0.2 in the last 2 years, Shanghai’s efficiency rose from around 0.3 in 2013 to 0.6 in 2014 to 1 in the last two years, Wuhan’s efficiency in the first year was 1, but by 2016 had fallen to 0.3, and Xian’s efficiency in the first three years was 1 but had also fallen to 0.3 by 2016.

Changchun, Changsha, Chengdu, Chongqing, Guiyang, Harbin, Hangzhou, Huhehot, Kunming, Lanzhou, Nanchang, Nanjing, Shenyang, Shijiazhuang, Taiyuan, Tianjin, Xining, Yinchuan, and Zhengzhou all had second stage efficiencies lower than 0.7 in all four years, of which Changchun, Harbin, Hangzhou, Nanchang, Shijiazhuang, Taiyuan, Tianjin, and Zhengzhou had maximum second stage efficiencies of only 0.4, and 0.2 was the highest efficiency achieved by Shijiazhuang and Zhengzhou. Therefore, there was a significant need for improvement in the second stage efficiencies in most cities.

Changsha, Chongqing, Jinan, Lanzhou, Shanghai, and Zhengzhou all had rising second stage efficiencies, with Jinan and Shanghai experiencing the largest increases; Jinan’s rose to 0.2 in 2013 and continued to rise to 1 in 2016, and Shanghai’s rose to around 0.3 in 2013, to 0.6 in 2014 and to 1 in 2015 and 2016.

The second stage efficiencies in the other 13 cities declined. The efficiency in Beijing was 1 in 2013 and 2014, below 0.2 in 2015 and 2016, and only 0.1 in 2016. Wuhan’s efficiency in 2013 was 1, fell to nearly 0.6 in 2014, rose to 0.8 in 2015, but fell to its lowest point of 0.3 in 2016, and Guiyang’s efficiency was 0.7 in 2013 and continued to decline. 

Overall, the efficiencies in the production stage in every city were better than in the health treatment stage, and the differences between the city efficiencies in the production stage were smaller than in the health treatment stage. However, the efficiencies were falling in both stages in most cities.

### 4.5. Efficiencies and Rankings for GDP, Health Expenditure, Birth Rate, Respiratory Disease, Mortality Rate, and Media Reports from 2013 to 2016

Table 5 shows the efficiencies for each city’s inputs and outputs from 2013 to 2016. In the first stage labor and energy consumption input efficiencies, Guangzhou, Lhasa, and Urumqi had labor efficiencies of 1 in all four years, Xining had a labor efficiency of 1 in all years except 2014 when it fell to 0.4, Fuzhou, Jinan, Shanghai, and Yinchuan had labor efficiencies of 1 for two years, and Beijing, Changsha, Hebei, Nanjing, Wuhan, Zhengzhou, and Shenyang had labor efficiencies of 1 for one year.

Chengdu, Chongqing, Harbin, Kunming, Guiyang, Shijiazhuang and Taiyuan had poor labor efficiencies, with the maximum being below 0.7; for example, Chongqing’s efficiency was 0.4 in 2013 and 2014.

Beijing, Guangzhou, Lhasa, and Nanning all had energy consumption efficiencies of 1 for all four years, and Changchun, Fuzhou, Haikou, Hefei, Urumqi, Xian had efficiencies above 0.8. Changsha, Chongqing, Guiyang, Huhehot, Kunming, Lanzhou, Shenyang, Shijiazhuang, Taiyuan, Xining, and Yinchuan had energy consumption efficiencies below 0.6, with Taiyuan’s being the worst at below 0.1 in all years. Shijiazhuang’s highest energy consumption efficiency was only about 0.3, and the highest in Guiyang, Xining, and Yinchuan was around 0.4.

However, Changsha, Chongqing, Fuzhou, Haikou, Hefei, Urumqi, Jinan, Shanghai, and Shenyang had increasing energy consumption efficiencies. Jinan’s rose from around 0.6 in 2013 to 1 in 2016, and Shanghai’s rose slightly from 0.6 in 2013 and 2014 to 1 in 2015 and 2016. The other 19 cities had declining energy consumption efficiencies, with Wuhan and Zhengzhou’s being the largest, falling from 1 to 0.6 in 2016.

In the second stage health expenditure input efficiencies, Fuzhou, Guangzhou, Haikou, Hefei, Nanning, and Urumqi had health expenditure efficiencies of 1 in all four years, and Beijing, Shanghai, Wuhan, and Yinchuan had relatively good performances. Beijing had an efficiency of 1 in 2013 and 2014, which dropped significantly to below 0.3 in the following two years, Wuhan’s health expenditure efficiency was above 0.8 from 2013 to 2015 but fell in 2016 to around 0.3, Yinchuan’s efficiency was 0.8 in 2015, and in all other years was above 0.9, and Jinan’s efficiency was less than 0.6 in the first three years, but in 2016 was 1. 

The health expenditure efficiencies in the other 17 cities were low, with the highest being about 0.7. The worst performing city was Zhengzhou with an efficiency of no more than 0.2 across the four years, Nanchang’s highest efficiency was less than 0.3, Shijiazhuang also only had an efficiency close to 0.3 in 2014 but was below 0.2 in the other years, Tianjin had its highest efficiency in 2014 at nearly 0.6, but in the other three years it was below 0.1, and Changchun, Guiyang, and Harbin achieved 0.4 in the first two years after which the efficiencies continued to decline.

Therefore, in most cities, the health expenditure efficiency decreased, with the cities with the largest declines being Beijing and Wuhan. Cities with rising efficiencies were Changsha, Chongqing, Jinan, Shanghai, and Shenyang, with Jinan and Shanghai having the strongest improvements. Shanghai’s health expenditure efficiency rose from 0.4 in 2013 to 1 in 2015 and 2016.

As can be seen in Table 6, the first stage GDP output efficiencies were better than the labor efficiencies. There were 19 cities with GDP efficiencies of 1, and only 12 cities needed GDP efficiency improvements. The highest efficiencies were in Fuzhou, Haikou, Lanzhou, Urumqi, Xian, and Yinchuan, which were all close to 0.8. In 2014 Xining’s GDP efficiency was 1, but in all other years was below 0.7.

In the second stage birth rate and mortality rate output efficiency, the birth rate efficiencies in most of the 31 cities were relatively good, but Beijing, Chengdu, Chongqing, Harbin, Shanghai, and Tianjin still needed improvements.

However, except for Beijing, Fuzhou, Guizhou, Haikou, Hefei, Lhasa, Nanchang, Nanning, Shanghai, Urumqi, and Yinchuan, which all had mortality rate efficiencies of 1, many cities needed to improve their mortality rate efficiencies. Shenyang had the worst mortality rate efficiency with the highest being only 0.6, and Harbin’s mortality rate efficiencies in 2014 and 2015 were below 0.5, but in the other years were above 0.8.

Changsha, Chengdu, Harbin, Hangzhou, Jinan, Lanzhou, and Nanjing had improving mortality rate efficiencies, but the city with the largest efficiency decline was Shijiazhuang, which fell from 1 in 2013 to 0.6 in 2016.

As can be seen in Table 7, in the second stage media report input efficiency, Fuzhou, Guangzhou, Haikou, Hefei, Lhasa, Nanning, and Urumqi had media report efficiencies of 1 in all four years. However, Changchun, Chengdu, Harbin, Hangzhou, Huhehot, Nanchang, Nanjing, Shijiazhuang, Taiyuan, Tianjin, Xining, Yinchuan, and Zhengzhou had maximum efficiencies below 0.6. In particular, Hangzhou’s highest score was only 0.2 in 2016 and its lowest was 0.1 in 2014, Nanjing’s efficiency was only 0.1 for all four years, and Shijiazhuang’s highest efficiency was only 0.1. 

Changsha, Chongqing, Jinan, Lanzhou, Shanghai, and Zhengzhou had increased media report efficiencies; Jinan’s rose from 0.1 in 2013 to 1 in 2016, Shanghai’s rose from 0.3 in 2013 to 1 in 2016, and Changsha’s rose from 0.1 in 2013 to 0.7 in 2016. 

The media report efficiencies in the other 18 cities declined. Beijing, Guiyang, Kunming, and Wuhan had the largest declines; Beijing’s dropped from 1 in 2013 and 2014 to below 0.1 in 2016, Guiyang’s fell from 0.9 in 2013 to less than 0.2 in 2016, Wuhan’s fell from 1 in 2014 to below 0.3 in 2016, and Xian’s fell from 1 to slightly above 0.2 in 2016.

In the second stage respiratory disease output efficiency, Fuzhou, Guangzhou, Haikou, Lhasa, Nanning, Wuhan, Urumqi, Xian, and Yinchuan had respiratory disease efficiencies of 1 for all four years. However, Chengdu’s respiratory disease efficiency rose from a low of 0.4 in 2013 to 0.6 in 2016, Shenyang’s efficiency was between 0.6 to 0.7 in the first two years, but fell to 0.5 in 2015 and 2016, Harbin’s efficiency in 2014 and 2015 was below 0.5, but reached 1 in 2016, and Hangzhou’s efficiency rose from below 0.6 in 2013 and 2015 to finish at around 0.8 in 2016. 

Changsha, Chengdu, Harbin, Jinan, and Nanjing, however, had rising respiratory disease rate efficiencies, with Changsha and Jinan both rising from close to 0.7 in 2013 to 1 in 2016. The efficiencies in the other 19 cities declined, with the largest declines being in Zhengzhou and Shenyang, which fell from nearly 0.7 in 2013 to around 0.5 in 2016**.**

## 5. Conclusions 

This study used a two-stage dynamic model to evaluate the economic growth, labor, air pollutant and carbon dioxide emissions, health expenditure, media coverage, birth rate, mortality rate, and respiratory disease efficiencies in 31 Chinese cities from 2013–2016, from which the following conclusions were made:

(1) Only Guangzhou and Lhasa achieved overall efficiencies of 1 in all four years. Nanning’s efficiency in the first three years was 1, and the overall efficiency in the final year was above 0.8.
(2) Fuzhou, Haikou, Hefei, Wuhan, Urumqi, and Xian had overall efficiencies between 0.6 and 0.9; however, most of the other 23 cities had overall efficiencies below 0.6. Therefore, there was a significant need for overall efficiency improvements.
(3) Beijing, Changchun, Chengdu, Guiyang, Harbin, Haikou, Huhehot, Nanchang, Nanjing, Shijiazhuang, Taiyuan, Wuhan, Urumqi, Xian, Xining, Yinchuan, Zhengzhou all had reduced overall efficiencies.
(4) Guangzhou, Lhasa, Nanning, Shanghai, and Beijing had production stage efficiencies of 1 for four consecutive years, 24 cities had production stage efficiencies below 0.8., and Lanzhou, Shijiazhuang, Taiyuan, and Xining’s had production stage efficiencies below 0.4.
(5) Changchun, Chongqing, Guiyang, Hangzhou, Huhehot, Jinan, Kunming, Nanjing, Shanghai, Taiyuan, Xining has rising production stage efficiencies, with Jinan, Shanghai and Nanjing having the most significant increases.
(6) The differences between the city efficiencies were greater in the second health treatment stage than in the production stage. Fuzhou, Guangzhou, Haikou, Hebei, Lhasa, Nanning, and Urumqi had health treatment stage efficiencies of 1. However, Changchun, Changsha, Chengdu, Chongqing, Guiyang, Harbin, Hangzhou, Huhehot, Kunming, Lanzhou, Nanchang, Nanjing, Shenyang, Shijiazhuang, Taiyuan, Tianjin, Xining, Yinchuan, and Zhengzhou had health expenditure efficiencies below 0.7, which indicated that there was a significant need for improvement.
(7) Fuzhou, Guangzhou, Haikou, Hefei, Lhasa, Nanning, and Urumqi. Changchun, Chengdu, Harbin, Hangzhou, Huhehot, Nanchang, Nanjing, Shijiazhuang, Taiyuan, Tianjin, Xining, and Yinchuan had media report efficiencies of 1; however, Zhengzhou’s highest efficiency was below 0.6. Changsha, Chongqing, Jinan, Lanzhou, Shanghai, and Zhengzhou had increasing media report efficiencies; however, 18 cities including Beijing, Guiyang, Kunming, and Wuhan, had falling efficiencies.
(8) Fuzhou, Guangzhou, Haikou, Lhasa, Nanning, Wuhan, Urumqi, Xian, and Yinchuan had respiratory disease efficiencies of 1 for all four years, Changsha, Chengdu, Harbin, Jinan, and Nanjing had rising respiratory disease efficiencies; however, all other cities had falling efficiencies and Chengdu, and Shenyang had the lowest respiratory disease efficiencies.
(9) Eleven cities had mortality rate efficiencies of 1, Shenyang and Harbin had the worst performances, and the mortality rate efficiencies in Changsha, Chengdu, Harbin, Hangzhou, Jinan, Lanzhou, and Nanjing continued to improve.

This study believes that the measures adopted by the central and local governments should focus on the two goals of carbon dioxide emissions control and air pollutant emissions reduction.

(1) The governance of air pollutant emissions should be given priority, which could reduce respiratory diseases, and provide residents with a healthier, cleaner living environment.
(2) Depending on the regional characteristics, the industrial economic structure, and the energy structure, the industrial economy should be transformed to reduce air pollutant emissions.
(3) As different measures are used to reduce carbon dioxide emissions and air pollutant emissions, different governance methods need to be introduced.
(4) The media reporting on air pollution increased in the last three years and there have been some improvements in the efficiency of respiratory diseases and the mortality rate. Media reports can focus attention on the harm to resident health of air pollution. Therefore the government should encourage greater media reports on air pollution and carbon dioxide emissions.
(5) Both the media report channels and media report accuracy need to be enhanced to raise resident awareness of the health impacts of air pollution and encourage greater resident advocacy.

## Figures and Tables

**Table 1:** Comparison of previous studies and this study.

Previous Studies	This Study
Research on energy consumption and environmental efficiencies [4,5,7,8,9,10,12,13,14,15,16,17,18,19,20,21] Research on production and emissions reduction efficiencies (two-stage DEA): [8,54,55,56,57]	Application of a network DEA model with a production stage to analyze energy consumption and the environmental effects, and a second health treatment stage focused on media and health expenditure efficiencies and the impact on respiratory diseases, and birth and mortality rates.
Research on energy consumption and its impact on public health. [22,23,24,25,26,27,28,29,30,31,32,33,34,35,36,37,38,39,40]	
Research on the media and its impact on public health [41,42,43,44,45,46,47,48]; Or on the media and its impact on the environment. [49,50,51,52,53].	

**Table 2:** Input and output variables.

Stage	Input Variables	Output Variables	Link	Carry Over
Stage 1	Labor by person	GDP by 100 million CNY	AQI	Fixed assets by 100 million CNY
			CO_2_ by Tonnes	
	Energy consumption by 100 million Tonnes			
Stage 2	Health Expenditure by 100 million CNY	Birth rate by 100 percent		
	Media reports by piece	Respiratory Diseases by person		
		Mortality Rate by 100 percent		

**Table 3:** Overall efficiency by city from 2013–2016.

NO.	DMU	2013	2014	2015	2016
1	Beijing	1	0.966071	0.568576	0.519882
3	Changchun	0.56771	0.59287	0.525205	0.452684
4	Changsha	0.46863	0.617711	0.567253	0.729385
2	Chengdu	0.45818	0.531151	0.477386	0.393256
5	Chongqing	0.49366	0.547923	0.503629	0.597086
6	Fuzhou	0.89194	0.783355	0.791411	0.799663
7	Guangzhou	1	1	1	1
8	Guiyang	0.53676	0.438777	0.406006	0.295224
9	Harbin	0.49047	0.545641	0.476909	0.401379
10	Haikou	0.81105	0.773064	0.763226	0.738336
11	Hangzhou	0.43408	0.581317	0.462183	0.482793
12	Hefei	0.87532	0.86949	0.867725	0.830989
13	Huhehaote	0.54711	0.594756	0.556189	0.433941
14	Jinan	0.37818	0.413205	0.432103	1
15	Kunming	0.53772	0.448668	0.537868	0.377133
16	Lanzhou	0.41234	0.424914	0.309618	0.461499
17	Lhasa	1	1	1	1
18	Nanchang	0.49642	0.519604	0.47023	0.357532
19	Nanjing	0.44666	0.590123	0.528991	0.492481
20	Nanning	1	1	1	0.833347
21	Shanghai	0.54561	0.675416	1	1
22	Shenyang	0.43746	0.581865	0.386561	0.419874
23	Shijiazhuang	0.23473	0.267289	0.221671	0.196542
24	Taiyuan	0.34416	0.231793	0.312488	0.271433
25	Tianjin	0.45335	0.524893	0.431113	0.453683
26	Wuhan	0.91577	0.67796	0.770985	0.492876
27	Urumqi	0.84466	0.815528	0.844491	0.7986
28	Xian	0.82364	0.810375	0.804485	0.441491
29	Xining	0.32835	0.392896	0.310533	0.262795
30	Yinchuan	0.59591	0.563451	0.451964	0.480645
31	Zhengzhou	0.47654	0.55585	0.524818	0.45244

**Table 4:** Two stage efficiencies by city from 2013–2016.

NO	DMU	2013-I	2013-II	2014-I	2014-II	2015-I	2015-II	2016-I	2016-II
1	Beijing	1	1	0.9321	1	0.9616	0.1755	0.924	0.1163
2	Changchun	0.7221	0.4133	0.7894	0.3963	0.7957	0.2547	0.735	0.1706
3	Changsha	0.7377	0.1996	0.7594	0.476	0.7588	0.3757	0.739	0.7198
4	Chengdu	0.5979	0.3184	0.603	0.4593	0.6423	0.3124	0.577	0.2097
5	Chongqing	0.4828	0.5046	0.4959	0.5999	0.5161	0.4911	0.509	0.6856
6	Fuzhou	0.7839	1	0.5667	1	0.5828	1	0.599	1
7	Guangzhou	1	1	1	1	1	1	1	1
8	Guiyang	0.3913	0.6822	0.4425	0.4351	0.4748	0.3372	0.422	0.1688
9	Harbin	0.7166	0.2643	0.7414	0.3499	0.7683	0.1856	0.649	0.1542
10	Haikou	0.6221	1	0.5461	1	0.5265	1	0.477	1
11	Hangzhou	0.74	0.1282	0.7707	0.3919	0.7923	0.1321	0.777	0.1884
12	Hefei	0.7506	1	0.739	1	0.7355	1	0.662	1
13	Huhehaote	0.5835	0.5108	0.7493	0.4402	0.7216	0.3907	0.623	0.2445
14	Jinan	0.5456	0.2108	0.5626	0.2638	0.561	0.3032	1	1
15	Kunming	0.4297	0.6457	0.4294	0.4679	0.5019	0.5739	0.441	0.3131
16	Lanzhou	0.3583	0.4663	0.3562	0.4936	0.3272	0.2921	0.35	0.573
17	Lhasa	1	1	1	1	1	1	1	1
18	Nanchang	0.7819	0.2109	0.7945	0.2447	0.7738	0.1667	0.606	0.1093
19	Nanjing	0.7275	0.1658	0.7119	0.4684	0.8723	0.1857	0.845	0.1398
20	Nanning	1	1	1	1	1	1	0.667	1
21	Shanghai	0.7509	0.3403	0.7301	0.6207	1	1	1	1
22	Shenyang	0.618	0.2569	0.7346	0.4291	0.5994	0.1737	0.534	0.3055
23	Shijiazhuang	0.345	0.1245	0.3507	0.1839	0.333	0.1103	0.321	0.0725
24	Taiyuan	0.324	0.3644	0.3152	0.1484	0.3192	0.3057	0.349	0.1943
25	Tianjin	0.7244	0.1823	0.7258	0.3239	0.7226	0.1396	0.723	0.1839
26	Wuhan	0.8315	1	0.7247	0.6312	0.7317	0.8103	0.715	0.271
27	Urumqi	0.6893	1	0.6311	1	0.689	1	0.597	1
28	Xian	0.6473	1	0.6208	1	0.609	1	0.567	0.316
29	Xining	0.2819	0.3748	0.3224	0.4634	0.2872	0.3339	0.348	0.178
30	Yinchuan	0.465	0.7268	0.4268	0.7001	0.3897	0.5142	0.4	0.5615
31	Zhengzhou	0.826	0.127	0.9141	0.1976	0.9151	0.1345	0.705	0.1995

**Table 5:** Labor, Energy consumption and Health expenditure efficiencies.

No.	DMU	2013 Labor	2014 Labor	2015 Labor	2016 Labor	2013 com	2014 com	2015 com	2016 com	2013 health	2014 health	2015 health	2016 health
1	Beijing	1	0.8643	0.9233	0.8522	1	1	1	0.9948	1	1	0.294822	0.187468
2	Changchun	0.5678	0.7661	0.5914	0.621	0.87642	0.81276	1	0.8485	0.414976	0.380241	0.378144	0.110669
3	Changsha	0.8634	0.8888	0.8898	1	0.61202	0.63007	0.6279	0.478	0.34986	0.594687	0.57991	0.701469
4	Chengdu	0.5339	0.6064	0.5648	0.6186	0.66199	0.59958	0.71993	0.5351	0.398593	0.546369	0.374886	0.151894
5	Chongqing	0.359	0.389	0.4519	0.4294	0.6065	0.60285	0.58031	0.5877	0.42243	0.444167	0.422754	0.651711
6	Fuzhou	1	0.9119	0.9533	1	1	0.95066	1	1	1	1	1	1
7	Guangzhou	1	1	1	1	1	1	1	1	1	1	1	1
8	Guiyang	0.4405	0.4769	0.5032	0.6754	0.3421	0.40802	0.44636	0.2586	0.455913	0.356066	0.340662	0.199014
9	Harbin	0.4807	0.5442	0.5518	0.5206	0.95258	0.93852	0.98467	0.7766	0.374447	0.392117	0.335015	0.149616
10	Haikou	0.6234	1	1	1	1	0.93804	1	0.9998	1	1	1	1
11	Hangzhou	0.8109	0.8409	0.8472	0.9615	0.66908	0.70056	0.73727	0.5929	0.205776	0.683214	0.226503	0.20956
12	Hefei	0.6798	0.6509	0.6711	1	0.96784	1	1	1	1	1	1	1
13	Huhehaote	0.8179	0.8189	0.7938	0.9219	0.34905	0.6797	0.64953	0.3248	0.455719	0.438759	0.465092	0.299598
14	Jinan	1	0.6813	0.7913	1	0.65216	0.44386	0.55178	1	0.365888	0.522551	0.549934	1
15	Kunming	0.4667	0.4648	0.4724	0.5676	0.39276	0.39399	0.53134	0.3147	0.447233	0.426316	0.430825	0.204695
16	Lanzhou	0.7573	0.6138	1	1	0.40866	0.33189	0.45684	0.2952	0.516016	0.616441	0.465734	0.466886
17	Lhasa	1	1	1	1	1	1	1	1	1	1	1	1
18	Nanchang	0.5991	0.6048	0.6045	0.7346	0.96471	0.98422	0.94312	0.477	0.26142	0.228074	0.215155	0.155642
19	Nanjing	0.8575	0.8548	0.9027	1	0.59757	0.56893	0.84181	0.6902	0.273521	0.926966	0.325941	0.181369
20	Nanning	1	1	1	0.6277	1	1	1	1	1	1	1	1
21	Shanghai	0.8438	0.7607	1	1	0.65807	0.69949	1	1	0.406247	0.700169	1	1
22	Shenyang	0.6944	1	0.6661	0.6634	0.54162	0.46917	0.53276	0.4052	0.316613	0.395016	0.328336	0.505158
23	Shijiazhuang	0.4129	0.4078	0.3997	0.4569	0.27704	0.29362	0.26635	0.1842	0.173477	0.260023	0.151801	0.120036
24	Taiyuan	0.5235	0.501	0.511	0.6181	0.12445	0.12932	0.12747	0.0791	0.460772	0.110231	0.376407	0.31194
25	Tianjin	0.8183	0.815	0.8111	0.8718	0.63062	0.63671	0.63415	0.5752	0.079892	0.551268	0.124668	0.078514
26	Wuhan	1	0.8514	0.9652	0.8793	1	0.70046	0.83447	0.5903	1	0.906775	0.841116	0.2676
27	Urumqi	1	1	1	1	0.91271	0.85196	1	1	1	1	1	1
28	Xian	0.7799	0.7491	0.7691	0.7976	1	0.98908	1	0.8026	1	1	1	0.421206
29	Xining	1	0.4658	1	1	0.39378	0.17901	0.41818	0.3694	0.517694	0.647523	0.440701	0.311428
30	Yinchuan	0.8387	0.7484	1	1	0.40113	0.33884	0.37493	0.2537	0.969579	0.937875	0.821219	0.937923
31	Zhengzhou	0.6734	1	0.8303	0.76	0.97867	0.82813	1	0.6509	0.197241	0.203929	0.203469	0.16008

**Table 6:** GDP, Birth rate and Mortality rate efficiencies.

No.	DMU	2013 GDP	2014 GDP	2015 GDP	2016 GDP	2013 BirthRate	2014 BirthRate	2015 BirthRate	2016 BirthRate	2013 MoralityRate	2014 MoralityRate	2015 MoralityRate	2016 MoralityRate
1	Beijing	1	1	1	1	1	1	0.757484419	1	1	1	1	1
2	Changchun	1	1	1	1	1	1	1	1	1	0.943723508	0.625284333	0.835382776
3	Changsha	1	1	1	1	1	1	1	1	0.742955245	1	0.984775926	1
4	Chengdu	1	1	1	1	0.918861033	1	1	0.780217303	0.79060127	0.951850185	0.863431639	1
5	Chongqing	1	1	1	1	0.951782638	1	1	1	0.962060465	0.872350746	0.773479694	0.820685221
6	Fuzhou	0.82228	0.71867	0.71263	0.71394	1	1	1	1	1	1	1	1
7	Guangzhou	1	1	1	1	1	1	1	1	1	1	1	1
8	Guiyang	1	1	1	0.91153	1	1	1	1	1	1	0.946143145	0.882185
9	Harbin	1	1	1	1	1	1	1	0.755066346	0.799766909	0.529610854	0.482234688	0.946226596
10	Haikou	0.81063	0.69618	0.67863	0.65647	1	1	1	1	1	1	1	1
11	Hangzhou	1	1	1	1	1	1	1	1	0.745631273	0.930127465	0.656720357	1
12	Hefei	0.91843	0.90518	0.89303	0.74737	1	1	1	1	1	1	1	1
13	Huhehaote	1	1	1	1	1	1	1	1	1	1	0.745190239	0.845687805
14	Jinan	0.74652	1	0.85863	1	1	1	1	1	0.767720381	0.772086257	0.631470315	1
15	Kunming	1	1	1	1	1	1	1	1	1	0.984423272	0.930724034	0.848694684
16	Lanzhou	0.72184	0.8021	0.6448	0.68517	1	1	1	1	1	1	0.92039942	1
17	Lhasa	1	1	1	1	1	1	1	1	1	1	1	1
18	Nanchang	1	1	1	1	1	1	1	1	1	1	0.945321474	0.874513086
19	Nanjing	1	1	1	1	1	1	1	1	0.692688735	0.838225171	0.651000805	0.931542124
20	Nanning	1	1	1	0.84689	1	1	1	1	1	1	1	1
21	Shanghai	1	1	1	1	0.826131235	0.936424866	1	1	1	1	1	1
22	Shenyang	1	1	1	1	1	1	1	1	0.530799746	0.610364061	0.391268599	0.489137711
23	Shijiazhuang	1	1	1	1	1	1	1	1	1	0.934158293	0.959517496	0.709749048
24	Taiyuan	1	1	1	1	1	1	1	1	1	0.988717893	0.92030362	0.881012833
25	Tianjin	1	1	1	1	1	0.959712255	0.624023359	1	0.647679333	0.714204628	1	0.627398917
26	Wuhan	0.85582	0.93804	0.84256	0.97341	1	1	1	1	1	1	0.930233279	0.95055451
27	Urumqi	0.78172	0.75844	0.76277	0.71286	1	1	1	1	1	1	1	1
28	Xian	0.78574	0.77776	0.76246	0.7744	1	1	1	1	1	1	1	0.955074444
29	Xining	0.62676	1	0.62696	0.6701	1	1	1	1	1	0.99996963	0.999936029	0.844608919
30	Yinchuan	0.80008	0.82313	0.69776	0.73413	1	1	1	1	1	1	0.999975909	0.994048643
31	Zhengzhou	1	1	1	1	1	1	1	1	0.861655916	0.864603189	0.804214815	0.784575919

**Table 7:** Media Report and Respiratory Disease efficiencies.

No.	DMU	2013 Media	2014 Media	2015Media	2016 Media	2013 Respiratory Disease Rate	2014 Respiratory Disease Rate	2015 Respiratory Disease Rate	2016 Respiratory Disease Rate
1	Beijing	1	1	0.1200693	0.0481249	1	1	0.92497012	0.960088535
2	Changchun	0.4119315	0.4421133	0.2585246	0.2622521	0.998974455	0.943723587	0.625284267	0.885976292
3	Changsha	0.1246329	0.3572711	0.1790275	0.7380899	0.690722237	1	0.98477591	1
4	Chengdu	0.4216476	0.521108	0.3844193	0.3762691	0.442522071	0.561862547	0.491011701	0.613341175
5	Chongqing	0.6336617	0.8671817	0.7162922	0.8965782	0.951625717	0.848862099	0.747757358	0.79196518
6	Fuzhou	1	1	1	1	1	1	1	1
7	Guangzhou	1	1	1	1	1	1	1	1
8	Guiyang	0.918545	0.514077	0.3579579	0.1593697	0.978036461	1	0.946168569	0.932664693
9	Harbin	0.2272614	0.5271841	0.1642169	0.213588	0.785220137	0.529610902	0.482234653	1
10	Haikou	1	1	1	1	1	1	1	1
11	Hangzhou	0.1062129	0.190903	0.1114363	0.1956354	0.603421208	0.724221147	0.506159525	0.773267118
12	Hefei	1	1	1	1	1	1	1	1
13	Huhehaote	0.575989	0.4416678	0.44912	0.2322561	0.970118394	0.999997497	0.745209475	0.891970769
14	Jinan	0.1302757	0.0998885	0.2055405	1	0.701032741	0.689093944	0.631470282	1
15	Kunming	0.8554831	0.5192394	0.7698739	0.4746839	0.973640658	0.984423315	0.930745805	0.896791168
16	Lanzhou	0.4167924	0.3708283	0.1494226	0.6790598	0.999600102	1	0.920421991	1
17	Lhasa	1	1	1	1	1	1	1	1
18	Nanchang	0.1646231	0.2613342	0.1302907	0.0777529	0.970285959	0.99999509	0.945350473	0.921624794
19	Nanjing	0.1282565	0.1108147	0.1319183	0.107396	0.671923477	0.838225242	0.651004435	0.971126508
20	Nanning	1	1	1	1	1	1	1	1
21	Shanghai	0.3368344	0.6165375	1	1	0.99107278	0.891035792	1	1
22	Shenyang	0.332968	0.6624735	0.1507739	0.3089423	0.676492155	0.693349047	0.471611461	0.51333815
23	Shijiazhuang	0.078617	0.1238988	0.0758856	0.0514374	0.962003685	0.934138526	0.945050382	0.743719581
24	Taiyuan	0.2746113	0.1888363	0.2675652	0.1014459	0.972572001	0.988717801	0.920336069	0.927092665
25	Tianjin	0.3714853	0.2361541	0.3013113	0.3881571	0.637374516	0.683509336	0.938712422	0.565893495
26	Wuhan	1	0.3556858	0.8171101	0.283407	1	1	1	1
27	Urumqi	1	1	1	1	1	1	1	1
28	Xian	1	1	1	0.2202027	1	1	1	1
29	Xining	0.2385705	0.279225	0.2270791	0.0766061	0.973330121	1	1	0.885261383
30	Yinchuan	0.484084	0.4623142	0.2072745	0.1872469	0.999871853	1	1	1
31	Zhengzhou	0.0747676	0.2091771	0.0993909	0.3222509	0.92686666	1	0.818143032	0.588318156
